# Characterization of the l-arabinofuranose-specific GafABCD ABC transporter essential for l-arabinose-dependent growth of the lignocellulose-degrading bacterium *Shewanella* sp. ANA-3

**DOI:** 10.1099/mic.0.001308

**Published:** 2023-03-15

**Authors:** Konstantinos Drousiotis, Reyme Herman, Judith Hawkhead, Andrew Leech, Anthony Wilkinson, Gavin H. Thomas

**Affiliations:** ^1^​ Department of Biology, University of York, PO Box 373, York, UK; ^2^​ Biology Technology Facility, University of York, PO Box 373, York. YO10 5YW, UK; ^3^​ Department of Chemistry, York Structural Biology Laboratory, University of York, PO Box 373, York. YO10 5YW, UK

**Keywords:** arabinose, solute transporter, sugars, *Shewanella*, ABC transporter

## Abstract

Microbes that have evolved to live on lignocellulosic biomass face unique challenges in the effective and efficient use of this material as food. The bacterium *Shewanella* sp. ANA-3 has the potential to utilize arabinan and arabinoxylan, and uptake of the monosaccharide, l-arabinose, derived from these polymers, is known to be mediated by a single ABC transporter. We demonstrate that the substrate binding protein of this system, GafA_Sw_, binds specifically to l-arabinofuranose, which is the rare furanose form of l-arabinose found in lignocellulosic biomass. The structure of GafA_Sw_ was resolved to 1.7 Å and comparison to *Escherichia coli* YtfQ (GafA_Ec_) revealed binding site adaptations that confer specificity for furanose over pyranose forms of monosaccharides, while selecting arabinose over another related monosaccharide, galactose. The discovery of a bacterium with a natural predilection for a sugar found abundantly in certain lignocellulosic materials suggests an intimate connection in the enzymatic release and uptake of the sugar, perhaps to prevent other microbes scavenging this nutrient before it mutarotates to l-arabinopyranose. This biological discovery also provides a clear route to engineer more efficient utilization of plant biomass components in industrial biotechnology.

## Introduction

The acquisition of carbon and energy sources is essential for all microbial cells to grow. In bacteria, sugars are known to be important sources of these nutrients, which must be first transported from the environment into the bacterium. These processes have been studied for decades in bacterial systems and many systems have been characterized in detail, demonstrating that a diverse range of systems can be used for the same substrates [1–3]. In the model bacterium *Escherichia coli* transporters are known for a wide range of different monosaccharides, with glucose being the preferred carbon source and then a range of others being used such as d-galactose, l-arabinose and d-xylose [4]. High-affinity transport can be mediated by ATP-binding cassette (ABC) transporters that use a substrate binding protein (SBP) to recognize the sugar with usually low or sub-micromolar affinity [5, 6]. Structures of these SBPs bound to different sugars have been known for many years and in every case the sugar adopts the pyranose (six-membered ring) form that is the most abundant in solution [6]. This includes structures of the d-galactose and l-arabinose SBPs, MglB and AraF, bound to d-galactopyranose and l-arabinopyranose, respectively [7–10].

A surprising discovery was made in 2009 with the finding that *E. coli* contains a second ABC transporter that handles both d-galactose and l-arabinose [11], although with lower affinity than either of the two well-known ABC systems. The crystal structure of the SBP, YtfQ, resolved this apparent paradox with the discovery that YtfQ bound d-galactofuranose (PDB: 2VK2) – the rare furanose form of the sugar that constitutes about 8 % of the d-galactose pool in solution (the other 92 % being d-galactopyranose that is recognized by MglB) [11]. This unique binding feature was confirmed by NMR experiments, and the coordinated expression of the *ytfQRTyjfF* and *mglABCD* operons by the galactose responsive transcription factors GalR and GalS suggested that *E. coli* expresses both transporters to capture all the free d-galactose present in the environment [11]. That study was hence the first to recognize that for a particular monosaccharide, there could be transporters specific for either the pyranose or the furanose forms, which due to their very different chemical shapes would dictate separate and distinct systems for each form. Shortly afterwards the discovery of a d-ribofuranose transporter [12] in bacteria already known to have a d-ribopyranose transporter supported this hypothesis, prompting a broader consideration of this question for other sugars found in nature [6].

One of the most abundant uses of furanose forms of sugars is in lignocellulose, specifically components of hemicellulose that contain l-arabinose, such as arabinoxylan, arabinan and rhamnogalacturonan-II [13–16]. In these complex polymers the l-arabinose is exclusively in the l-arabinofuranose (l-Ara*f*) form [17, 18] and after release from these glucans by the action of l-arabinofuranosidases [19] will be present in the l-Ara*f* form for a short time before spontaneous chemical interconversion to the pyranose form, which forms the vast majority (around 90%) at chemical equilibrium [20].

YtfQ is also able to bind l-arabinose, in fact with an apparent higher affinity than d-galactose, and later work has demonstrated that the operon *ytfQRTyjfF* is regulated by the l-arabinose responsive transcription factor AraC [21]. This led us to speculate that the uptake of l-Ara*f* in bacteria could be of physiological significance and we noted that the YtfQ homologue in *Mycobacterium smegmatis *, MSMEG_1712, was encoded with l-arabinose-degrading genes [11]. This hypothesis has been supported in an elegant study that demonstrated that MSMEG_1712 is an l-Ara*f* and d-Gal*f* binding protein [22]. The absence of this protein in the related pathogen *Mycobacterium tuberculosis* but its presence in the soil-dwelling *M. smegmatis* suggests a physiological function outside of the host in a soil environment rich in plant and fungal biomass-derived sugars [23]. Given the confirmed functions of YtfQ proteins we now propose to rename YtfQ and its homologues as GafA for their galacto- and arabinofuranose binding function and the *ytfQRTyjfF* operon to be *gafABCD* following nomenclature for other ABC transporters. In this study we identify GafA homologues in a range of bacteria and focus on the function of these systems in the use of lignocellulosic sugars. Specifically, we focus on two uncharacterized GafA proteins from soil bacteria, namely *Sinorhizobium meliloti* (GafA_Sm_) and the lignocellulosic degrader *Shewanella* sp. ANA 3 (GafA_Sw_), as published genetic evidence demonstrates that the latter bacterium is dependent on this uncharacterized transporter for growth with l-arabinose as a sole carbon source [24, 25].

## Methods

### Identification of *gafAs* in bacterial phyla

**Fig. 1.**
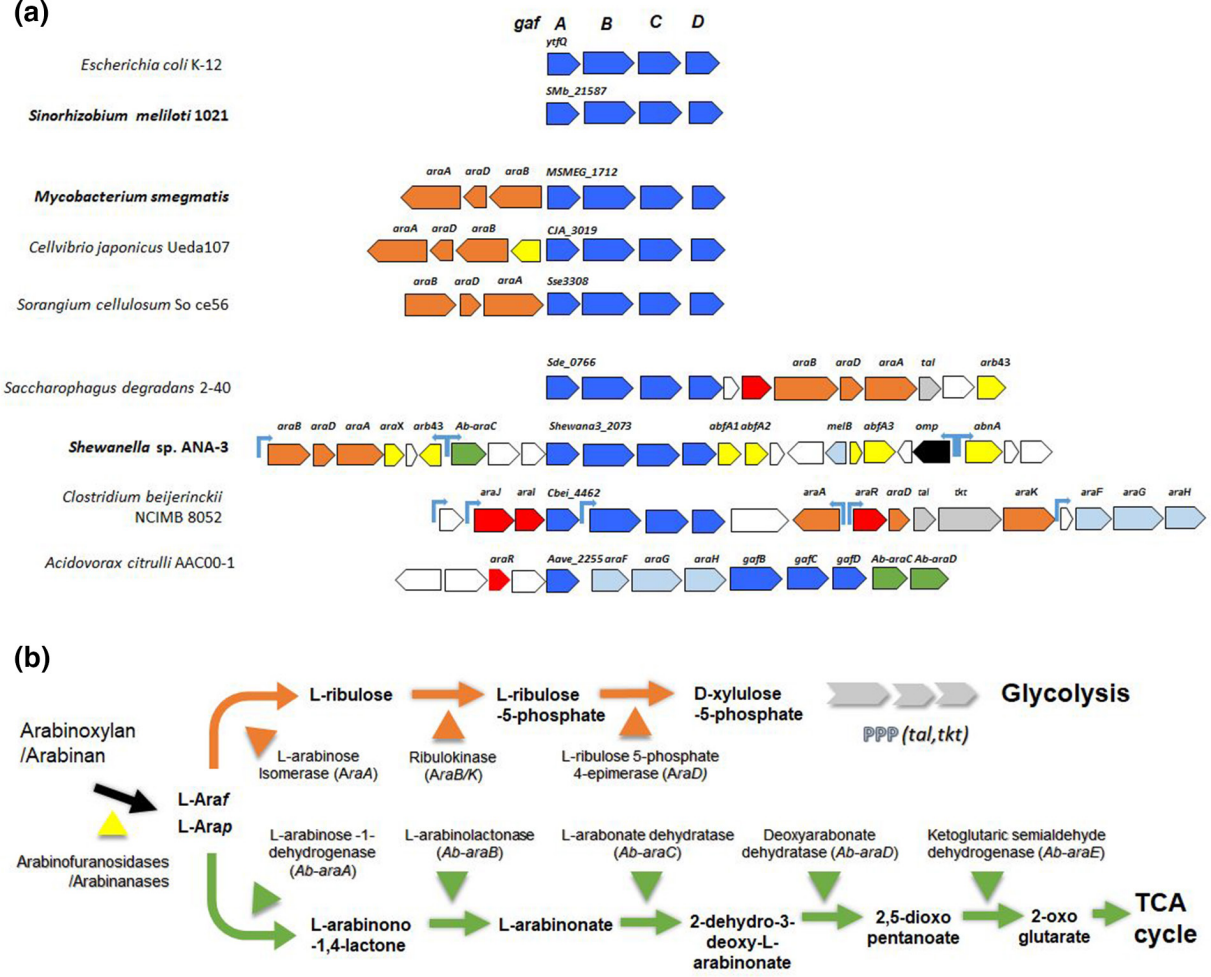
(a) Genetic context of *gafABCD* genes encoding known and candidate galacto- and arabino-furanose transporters, highlighting linkage to arabinose catabolic gene clusters in soil-dwelling microbes. The locus tag for the Gaf SBP subunit is indicated. Genes encoding Gaf ABC transporters are in blue, while those for other sugar ABC transporters are in light blue and a possibly related porin in black. Catabolic genes in the AraBAD pathway are in orange and the alternative *A. brasiliense* system in green. Known and probable transcriptional regulators are in red. Arabinases and l-arabinofuranosidases are in yellow, while genes encoding pentose phosphate pathway components are in grey. Genes in the clusters with no strong functional predictions are in white. The light blue arrows indicate promoters regulated by AraJ/R-like regulators (from RegPrecise). (**b**) Simple schematic showing the steps in the AraBAD (orange) and *A. brasiliense* (green) pathways for l-arabinose catabolism.

The repertoire of GafA proteins was explored initially using SEED viewer and MicrobesOnline [24], and organisms with interesting linked genes were identified, including some from biotechnologically important genomes (included in Fig. 1a). A larger dataset using the protein sequences of these YtfQ/GafA proteins was collected using blastp analysis. The lowest scoring GafAs from α, β, γ and δ proteobacteria as well as *Firmicutes *, *Spirochaetes* and *Actinobacteria *, which still presented at least 45 % or higher identity to the query protein sequence, were inputted on new blastp searches. The fresh searches were intended to collect more GafAs and expand the collection by restricting the search for each bacterial orthologue to its respective phylum or class. The process was repeated until previous results were observed again so that a non-redundant set of GafAs was produced. To avoid creating large and impractical clades, only one bacterium from each species was included in the downstream phylogenetic analysis. The identified protein sequences, which amounted to 110, were used to produce a phylogram as described below.

### Phylogenetic tree reconstruction

Multiple sequence alignments and homology searches were performed using the online sequence analysis software mafft [26] using the l-iNS-I refinement method or Clustal Omega [27]. The sequence alignments were curated in the Gblocks 0.91b tool of the phylogeny.fr [28]. The alignments were inputted into PhyML 3.0 [29] for automatic model selection using the Akaike information criterion (AIC). The AIC estimates the relative quality of statistical models and chooses one based on the quantity of information lost when a given method is used to represent the process that generated the data. The substitution model parameters as calculated by the AIC are described in Table 1 and their use in the phylotrees constructed is designated. Reconstruction of the phylograms was performed in PhyML 3.0 using the maximum-likelihood method based on the substitution model and the parameters calculated by the AIC. The confidence of the branches was inferred using 500 bootstrap replications. The resulting newick files were inputted into the Interactive Tree Of Life (iTOL) to display and annotate the trees (http://itol.embl.de) [30]. Further annotations were made in the scalable vector graphics editor, Boxy SVG.

**Table 1.** Data collection and refinement statistics

	Gaf_SW_- *l* -Ara*f* (5OCP)		
Diffraction Source, λ (Å)	DLS i03, 0.9795	Refinement*	
Space group	P2_1_2_1_2_1_	Refinement programme	refmac 5.8.0158
a, b, c (Å)	73.92, 86.33, 87.28	Resolution range (Å)	43.45–1.70
α, β, γ (°)	90.00, 90.00, 90.00	No. of reflections (working set)	58 975 (4272)
Resolution range (Å)*	43.64–1.70 (1.73–1.70)	No. of reflections (test set)	3081 (231)
Total Reflections	62122	Completeness	99.9 (99.7)
*R*_merge_	0.082 (0.597)	Final *R*_work_	0.167 (0.218)
I/σ(I)	3.77	Final *R*_free_	0.204 (0.261)
Data redundancy	7.4 (7.1)	Wilson B-factor (A^2^)	18.0
		Number non-H ofatoms	
		Protein	4618
		Ligand	92
		Solvent	461
		RMS deviations	
		Bond lengths (Å)	0.02
		Bond angles (°)	2.03
		Average B factors (Å^2^)	
		Protein	23.36
		Ligand	33.05
		Solvent	34.85
		Ramachandran plot (favoured/ allowed/outlier)	93.0/6.6/0.4

*Values in parentheses refer to the outer resolution shell 1.73–1.70 Å.

Functional annotations of the genes involved in the arabinose, galactose and xylose metabolism and related pathways were derived using Microbes Online (http://www.microbesonline.org/); PATRIC 3. 4. 2 (https://www.patricbrc.org) and Biocyc (https://biocyc.org/).

### Cloning of substrate binding proteins

The gene sequence for *shewana3_2073* was codon optimized in JCat and synthetically produced by IDT. The synthetic gene was cloned into pET20b vector using Gibson assembly. The *smb21587* gene sequence was amplified from genomic DNA of *S. meliloti* 1021 using Q5 DNA polymerase (NEB) with the following primers: GafASmF CATGCCATGGCCGAACTCGTCGTCGGCTTT and GafASmR CCGCTCGAGGTAGCCGAGGCCTTTCTTTTCTTCG. The PCR product was digested with *Nco*I and *Xho*I to allow cloning into pET20b. The sequence of the cloned genes was confirmed by DNA sequencing (Sigma Oligo) using the T7 and T7 terminator primers.

### Expression and purification of SBPs

Expression of the proteins was optimal at 20 °C after 20 h of growth following induction with 1 mM IPTG. Expression was performed with cells grown either in Lysogeny broth (LB) or Terrific broth (TB). The periplasmic fraction was isolated by osmotic shock [31] and loaded in the Ni-nitrilotriacetic acid column for purification. Fractions were pooled and dialysed in PBS. Protein levels were quantified on an Epoch Microplate Spectrophotometer.

For preparation of ligand-free protein, the filtered periplasmic extract was injected into a pre-equilibrated His-tag column. The proteins were washed with a decreasing gradient of guanidine hydrochloride (4–0.5 M) in washing buffer (i.e. 50 mM Tris, 200 mM NaCl and 25 mM imidazole, pH 7.5). The column was further treated with refolding buffer (i.e. washing buffer mixed with 500 mM arginine monohydrochloride, 4 mM reduced glutathione and 0.4 mM oxidized glutathione, pH 7.5). Finally, the protein was eluted using elution buffer (i.e. washing buffer mixed with 20 % glycerol and 500 mM instead of 25 mM imidazole, pH 7.5).

### Circular dichroism (CD)

Spectra were obtained using a J-810 spectropolarimeter (Jasco) controlled by the supplied software SpectraManager version 1.53.00 (Jasco). Proteins were dialysed into 50 mM NaF and 20 mM Tris-HCl pH 7.5 and diluted to a concentration of 10 µM. The spectrum was recorded at 20 °C (Peltier temperature controller) in a 1 mm pathlength quartz cuvette (Starna) between 180 and 240 nm at 100 nm min^–1^ with 1 nm pitch. The molar elipticity (θ) data obtained were corrected by subtracting the buffer control and were plotted against the wavelength (nm) in GraphPad Prism 7.0.

### Crystallization and structural determination of GafA_Sw_

The Hydra-96 Microdispenser (Robbins Scientific) and Mosquito Crystal (TTP Labtech) robots were used to dispense commercially sourced crystallization solutions and the protein–ligand solution containing GafA_Sw_ with l-arabinose. The crystallization screens were performed in the vapour-diffusion sitting–drop format with a mix of 150 nl crystallization solution and 150 nl GafA_Sw_ and l-arabinose at final concentrations of 8 mg ml^−1^ and 1.25 mM, respectively. The crystallization conditions that produced the crystal used to resolve the structure of this protein was 0.2 M ammonium nitrate, pH 6.2, and 20 % PEG 3,350 (B7 of the PEG/ION HT tray; Hampton Research). This crystal was harvested from the sitting–drop and coated in a solution of the aforementioned crystallization solution supplemented with glycerol as the cryo-protectant, to a final concentration of 20 % (v/v).

Diffraction data were collected to a resolution of 1.7 Å using the macromolecular crystallography (MX) Beamline I03 at the Diamond Light Source, Hartwell Science and Innovation Campus, and processed with dials. The space group and cell dimensions (Table 1) were consistent with two GafA_Sw_ chains in the asymmetric unit giving a Matthews coefficient of 2.1 Å^3^ Da^–1^ and a solvent content of 42.04 %. The CCP4i2 suite of programs was used to scale the data and resolve the structure. The processed dials data were scaled using aimless, pointless, Ctruncate and FreeRflag [32, 33]. The structure was resolved by molecular replacement using molrep [34, 35] using the coordinates for an *E. coli* d-galactofuranose binding protein, YtfQ (PDB: 2VK2), as the search model. As expected two solutions were obtained. The initial model built by molrep was then refined using iterative cycles of refmac5 [35] followed by manual model building in coot [36] until convergence was reached. The final structure was then deposited with the PDB ID 5OCP via wwPDB [37].

For structural comparisons between the furanose binding proteins we included the GafA_Ms_ bound to l-Ara*f* (PDB: 6HBM). This has two different binding positions for l-Ara*f* in the two monomers in the asymmetric unit. The terminal carbon of the ligand in one monomer is next to Glu28 and the other is next to Asp105, suggesting that the ligand is rotated 180° about the plane of the furan ring. Using our structure of GafA_Sw_ bound to l-Ara*f* (PDB: 5OCP), we determined that the probable orientation of l-Ara*f* in GafA_Ms_ is in chain A of the PDB structure with the terminal carbon being coordinated by Asp105, which is more consistent with the l-fucofuranose (PDB: 6HYH) and l-galactofuranose (PDB: 6HBD) bound structures. Hereafter, we used chain A from 6HBM for structural comparisons.

### Differential scanning fluorimetry (DSF)

Protein–ligand solutions were dispensed into the wells of a 96-well thin wall PCR plate (ie. Genomic Fast Optical 0.1 ml plates). Each well contained 3–5 µM protein, 0.1× (2.5 µl of 0.8×) SYPRO orange and appropriate volumes of ligands. Different stocks of the ligands were prepared, so that 2 µl was transferred in each well to reach desired concentrations of 0.6, 6, 60, 600 and 1200 µM with four repeats of each ligand concentration for each protein. A final well volume of 20 µl was filled up by the protein’s buffer. For each run, there were eight reference wells where ligands were excluded. Also, four reference wells were included that contained only buffer. The plates were sealed with optical sealing tape (Bio-Rad). The instrument run was set as instructed in the manual (Applied Biosystems). The plate was heated to 99 °C in increments of 0.5 °C. Data analysis was undertaken using Microsoft Excel. Δ *T*_m_ calculations were made by subtracting the *T*_m_ of the mean protein-only well with the data with added ligands. We note that the ratio of the furanose/pyranose forms of the monosaccharides will probably change slightly during the temperature ramp, although this should not impact on the conclusion of the experiment profiling potential ligands.

### Fluorescence spectroscopy

Tryptophan and tyrosine fluorescence spectroscopy was performed using a FluoroMax 4 fluorescence spectrometer (Horiba Jobin-Yvon) with a water bath for temperature control. The maximum emission for each protein was determined by spectral analysis, i.e. excitation at wavelengths of 280, 295 and 297 nm at slit widths equal to 3 nm. Kinetic experiments for quantification of the binding affinities were performed with purified protein at concentrations ranging from 0.5 to 1.5 µM in PBS, pH 7.5. Total volume of the sample was 3 ml, which was excited at 280 nm for GafASm and 297 nm for GafASw with slit widths of 3 nm. Emission was monitored at 330 (GafASw) or 342 nm (GafASm) with slit widths of 3 nm. The fluorometer operated in a time-based acquisition mode with a run time of 360–500 s and an integration time of 1 s. Increasing concentrations of ligand were added to the protein solution and fluorescence change was monitored. The cumulative fluorescence change for each timepoint was plotted against the cumulative concentration of ligand in SigmaPlot 11 and the *K*_D_ was calculated from the hyperbolic fit of the binding curve.

### Isothermal titration calorimetry

Calorimetry experiments were performed in the VP-ITC instrument (MicroCal, GE Health Sciences). Proteins and ligands were dissolved in the same buffer in each experiment (i.e. PBS or NaCl). The concentration of the protein in the cell ranged from 50 to 120 µM, according to the *c* value, where *c*=[protein]/(predicted) *K*_D_. The ligand concentration in the syringe was in 10- or 7-molar excess compared to the protein. Experiments were carried out in PBS or Tris-HCl (NaCl), pH 7.5 at 25 °C. The solutions in the cell and syringe were both degassed at 20 °C for 5 min before use. A typical run included 26 titrations, each one delivering 10 µl s^−1^ ligand with 240 s delay between injections. The acquired raw titration data were analysed in MicroCal Origin 7 software where binding isotherms were fitted by an iteration process using the one-set of sites model.

## Results

### Gaf ABC transporters are present in diverse bacteria and often co-occur with arabinan/l-arabinose catabolic genes

To extend the study of Gaf ABC transport systems beyond the characterization of GafA_Ec_ (YtfQ), we identified around 100 homologues in other bacteria using SEED and MicrobesOnline [24, 38]. These were found in several biotechnologically important bacteria and plant symbionts with representatives from diverse phyla (Fig. 1a). While some resembled the *E. coli* system, in not being linked to any related catabolic genes, it was notable that many orthologues were encoded within larger clusters containing genes involved in l-arabinose catabolism, most often the classical *araBAD* encoded route via l-ribulose (Fig. 1b). This included the previously noted *M. smegmatis* system and other bacteria that are likely to encounter l-arabinose in their soil environment, such as *Cellvibrio japonicus* Ueda 107 (γ-proteobacteria) and *Sorangium cellulosum* So ce56 (δ-proteobacteria). More complex examples were also seen in the γ-proteobacteria *Saccharophagus degradans* and *Shewanella* sp. ANA-3, as well as *Clostridium beijerincki* NCIMB 8052 (* Firmicutes *) and *Acidovorax citrulli* AAC00-1 (β-proteobacteria). These bacteria contain extended catabolic clusters including genes encoding secreted l-arabinofuranosidases (yellow symbols in Fig. 1) and in some cases genes for an alternative oxidative non-phosphorylative arabinose catabolic pathway discovered in *Azospirillum brasiliense* that produces 2-oxo-glutarate [39] (Fig. 1b). Intriguingly the *Clostridium beijerinckii* cluster also contains genes encoding a typical l-arabinopyranose type ABC transporter (*araFGH*) as well as the putative GafABCD system suggesting an evolutionary advantage to expressing transporters together to access all available l-arabinose, and in *Acidovorax citrulli* AAC00-1 the pyranose transporter genes interpose with the genes for the predicted furanose transporter (Fig. 1a). It is notable that there is no direct link to any d-galactose catabolic genes, while linkage to l-arabinose catabolism is found in many cases in strongly suggestive biological contexts.

**Fig. 2.**
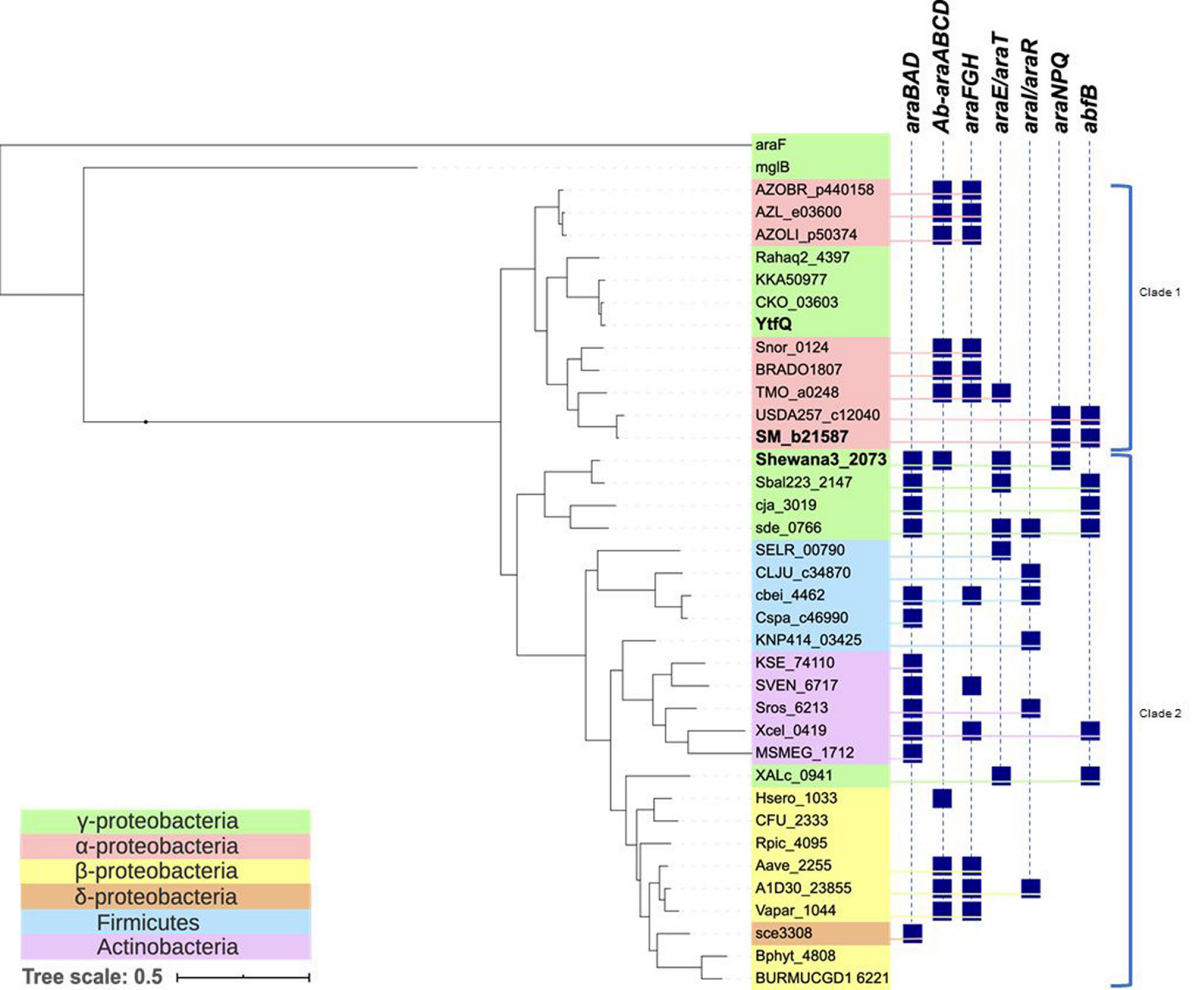
Phylogeny of GafA proteins. Maximum likelihood analysis of 36 selected GafA proteins, with AraF and MglB as outgroups, with the taxonomic position of the organisms coloured according to the inset key. To the right is a mapping of presence/absence of genes related to arabinose transport and utilization (±20 kb of the *gafA* gene). Genes/operons code for: *araBAD*=primary arabinose catabolism, *Ab-araABCD*=arabinose catabolic pathway III from *A. brasiliense*, *araFGH*=arabinopyranose ABC transport, *araE/araT*=arabinose secondary transport, *araI/araR*=arabinose, *araNPQ*=arabinosides ABC transport, *abfB*=α-l-arabinofuranosidase. Protein names are based on locus tags and can be identified in UniProt.

Examining the GafA protein sequences themselves and their phylogenetic relationships (Fig. 2), the sequences fall into two nominal clades, when using the related AraF and MglB proteins, which bind l-arabinopyranose and d-galactopyranose, respectively, as outgroups. When the presence of other genes in the same cluster are added, the split into Clade I and Clade II is reinforced, particularly by the *araBAD* genes, encoding the classical pathway to l-arabinose catabolism, which are exclusively seen linked to Clade II GafA systems (Fig. 2). While other genes relating to l-arabinose catabolism are more prevalent in Clade II clusters, this grouping is not exclusive, with some linkage to Clade 1 clusters. Also, when the phylogeny of the organisms is overlaid on the tree (Fig. 2), clade I contains only sequences from proteobacteria (α and γ proteobacteria) and includes GafA_EC_ (YtfQ) that binds d-galactofuranose in addition to l-Ara*f*. Together these *in silico* data suggest that in some bacteria GafA might have evolved to confer not just specificity for furanose forms of common monosaccharides, but also selectively for l-Ara*f*.

### Characterization of GafA proteins previously implicated in l-arabinose uptake

The genome context analysis revealed multiple Gaf systems encoded along with other genes related to l-arabinose catabolism. To help direct the experimental study, we sought further bioinformatic and experimental support before deciding which systems to characterize biochemically. The RegPrecise tool that predicts regulons in diverse bacteria was used to investigate predicted arabinose-induced regulons in the aforementioned bacterial candidates [40]. In *Clostrdium beijerinki* NCIMB 8052 there is a predicted AraR regulon with binding sites at six locations, all contained within the large gene cluster identified in Fig. 1(a). A similar prediction for the *Shewanella* sp. ANA-3 AraR regulon identifies five locations, again all within the large cluster indicated in Fig. 1(a), strongly supporting the hypothesis that these Gaf transporters are encoded as part of l-arabinosespecific regulons.

We then used the Fitness browser of MicrobesOnline to investigate the effect of *gafA* disruption caused by transposon insertion [24]. The deletion of *shewana3_2073* caused a notable decrease in fitness of *Shewanella* sp. ANA-3 when grown on l-arabinose as the sole carbon source [25]. Related data from Rodionov *et al*. [41] showed that *Shewanella* sp. ANA3 is unable to use galactose as the sole carbon source, consistent with a lack of a likely secondary transporter for galactose uptake, i.e. GalP. Together these data suggest a role for the Gaf system in l-arabinose utilization. Another example examined was the *gaf* system from *Sinorhizobium meliloti *. There is independent evidence that expression of the genes encoding this uncharacterized system are induced by the presence of l-arabinose, l-fucose and d-talose in the growth media [42]. Based on the evidence above, we chose Smb_21587 (GafA_Sm_) from *Sinorhizobium meliloti* and Shewana3_2073 (GafA_Sw_) from *Shewanella* sp. ANA-3 to analyse further, representing examples from Clade I and Clade II (Fig. 2), both of which have published supporting experimental evidence.

Coding sequences were cloned into pET20b and the recombinant proteins were expressed in *E. coli* as fusion proteins with a C-terminal hexahistidine tag, which were then purified using nickel affinity chromatography (Fig. S1A, available in the online version of this article). To remove pre-bound ligand and hence make the protein suitable for measuring ligand binding, the proteins were treated to an on-column unfold/refold step as used previously for GafA_Ec_ and other SBPs [11, 43]. To confirm the protein had refolded, the overall structure was assessed using CD analysis, demonstrating a similar pattern of secondary structure for the refolded and native proteins (Fig. S1B).

### GafA_Sm_ is a typical GafA protein binding both d-galactose and l-arabinose

To study the ligand binding range of a Clade I GafA with a simple gene layout (Figs 1a and 2), we used a DSF method[44], where the binding of a ligand is reflected by the thermal stabilization of the protein. The addition of l-arabinose produced the greatest increase in melting temperature (Δ*T*_m_ of +14.4 °C at 1.2 mM ligand) while d-galactose and d-fucose also produced pronounced shifts (Δ*T*_m_ of +11.6 and +11.8 °C respectively, at 1.2 mM ligand) (Table 2, Fig. S3A), a pattern very similar to that observed previously with GafA_Ec_ [11]. Consistent with the binding profile of GafA_Ec_, the addition of d-talose and d-allose to GafA_Sm_ produced small Δ*T*_m_ values and d-glucose failed to cause a change in thermal stability, even at higher concentrations (data not shown). The latter could be attributed to the negligible concentrations of the furanose form of glucose (<0.5 %) due to its unstable nature, which differs from the other sugars that can be up to around 30 % in the furanose form at equilibrium (Table 2 and Fig. S2). We conclude the GafA_Sm_ has similar properties to GafA_Ec_, i.e. it binds to both d-galactose and l-arabinose and probably recognizes both of these sugars during its normal function, which is probably a conserved feature of Clade I GafA proteins that are not usually genetically linked to l-arabinose catabolic genes.

**Table 2.** DSF data for GafA_Sm_ and GafA_Sw_ NC indicates no apparent change in Δ*T*_m_ upon ligand addition. Ratios of furanose/pyranose are taken from literature values at the indicated temperatures [20].

Monosaccharide	Furanose/pyranose ratio (%) at indicated temp.	Δ*T*_m_ (°C) at 1.2 mM [ligand]	
		GafA_Sm_	GafA_Sw_
l-Arabinose	8.5/91.5 (31 °C)	14.4±0.2	6.5±0.2
d-Galactose	6/94 (31 °C)	11.6±0.2	NC
d-Fucose	5/95 (31 °C)	11.8±0.2	2.1±0.2
d-Allose	8.5/91.5 (31 °C)	1.0±0.1	5.0±0.1
d-Talose	29/71 (28 °C)	3.4±0.2	NC
d-Glucose	0.3/99.7 (31 °C)	NC	NC

### GafA_Sw_ binds l-arabinose selectively over d-galactose

The GafA protein from Shewanella sp. ANA-3 sits in Clade II and its ligand binding specificity was similarly investigated using DSF analysis (Fig. S3 and Table 2). Again, l-arabinose gave the greatest increase in thermal stability of 6.5 °C, suggesting that it binds with the highest affinity. d-Allose caused the second highest change in thermal stability (Δ*T*_m_ of +4.9 °C). The addition of d-fucose, another binder of GafA_Ec_ and GafA_Sm_, resulted in a weaker increase in *T*_m_ (2.1 °C). We also found that d-talose, which could be weakly bound to GafA_Ec_ and GafA_Sm_, did not result in any changes in thermal stability of GafA_Sw_. However, most significantly, d-galactose did not cause any detectable thermal shift, a unique feature of GafA proteins examined to date.

**Fig. 3.**
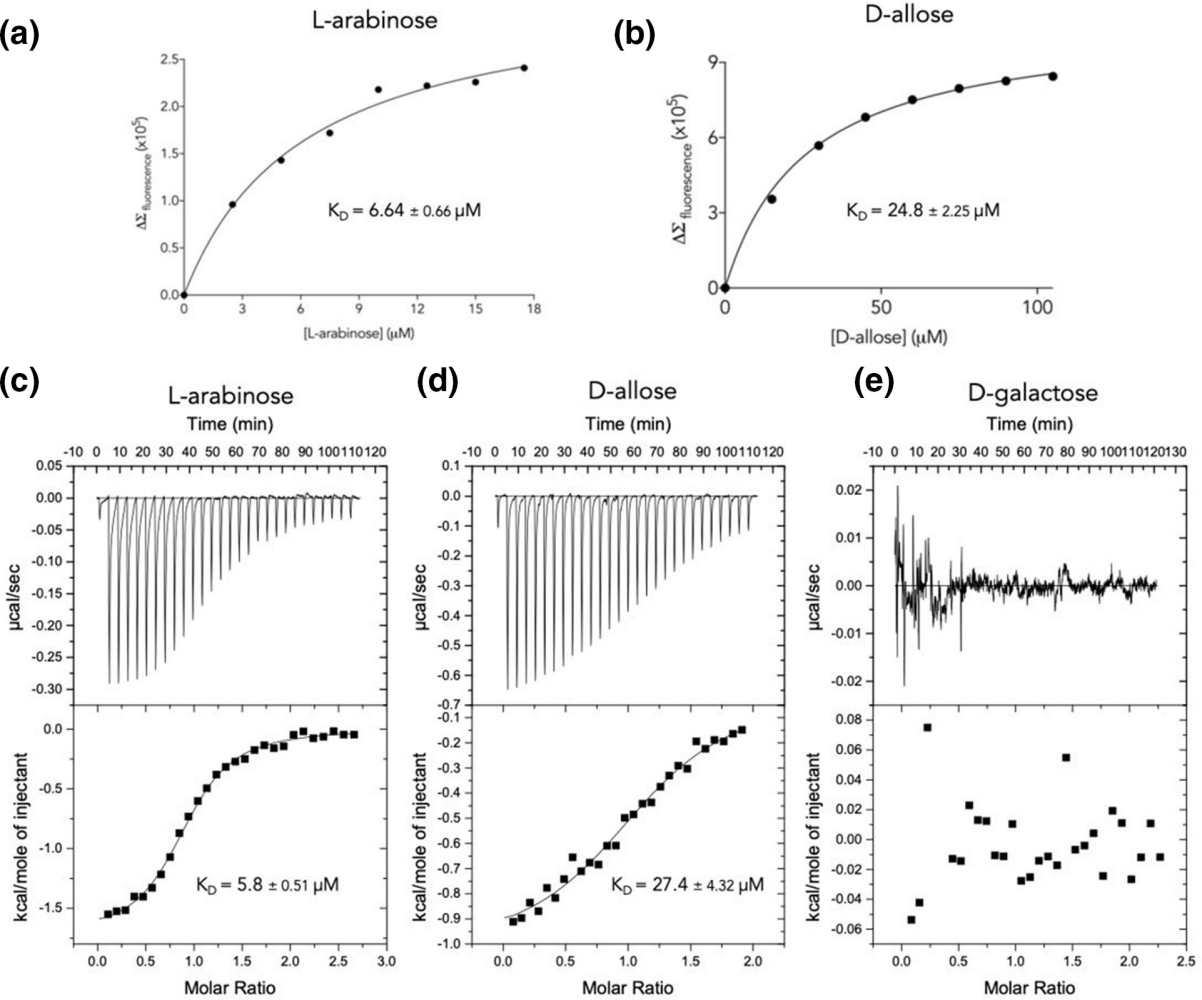
Intrinsic fluorescence and ITC analysis of ligand binding to GafA_Sw_. Plot of fluorescence changes against cumulative concentration of (a) l-arabinose and (b) d-allose . The binding affinity (*K*_D_) was calculated using the one-site specific binding model. ITC experimental data for the binding of (c) l-arabinose, (d) d-allose and (e) d-galactose to GafA_Sw_. The top panel (thermogram) represents the heat differences upon each injection of ligand and the lower panels (isotherm) show integrated heats of injection (■). The best fit (solid line) was calculated using the one-site binding model using the Microcal Origin software. Affinity was calculated using data from three different replicates for each ligand.

To validate these initial binding data using a more quantitative method, we assessed the binding of d-allose, l-arabinose and d-galactose to GafA_Sw_ using intrinsic fluorescence spectroscopy and isothermal titration calorimetry (ITC). First, the changes in the intrinsic fluorescence of GafA_Sw_ in the presence of a ligand were titrated with increasing concentrations of the various ligands. Through this assay, the *K*_D_ value for l-arabinose binding was determined to be 6.64±0.66 µM, with that for d-allose being 24.8±2.25 µM (Fig. 3a), consistent with the magnitude of the changes seen by DSF. These findings were further validated using ITC with the *K*_D_ calculated for l-arabinose and d-allose binding being 5.8±0.51 and 27.4±4.32 µM, respectively (Fig. 3b). Again, we were unable to detect any changes in intrinsic fluorescence when d-galactose was added to the protein at any concentrations, nor detect binding by ITC (Fig. 3e). Hence, GafA_Sw_ appears to have unique features in selecting l-arabinose over d-galactose that might relate to its biological function.

### The structure of GafA_Sw_ reveals bound l-arabinofuranose

**Fig. 4.**
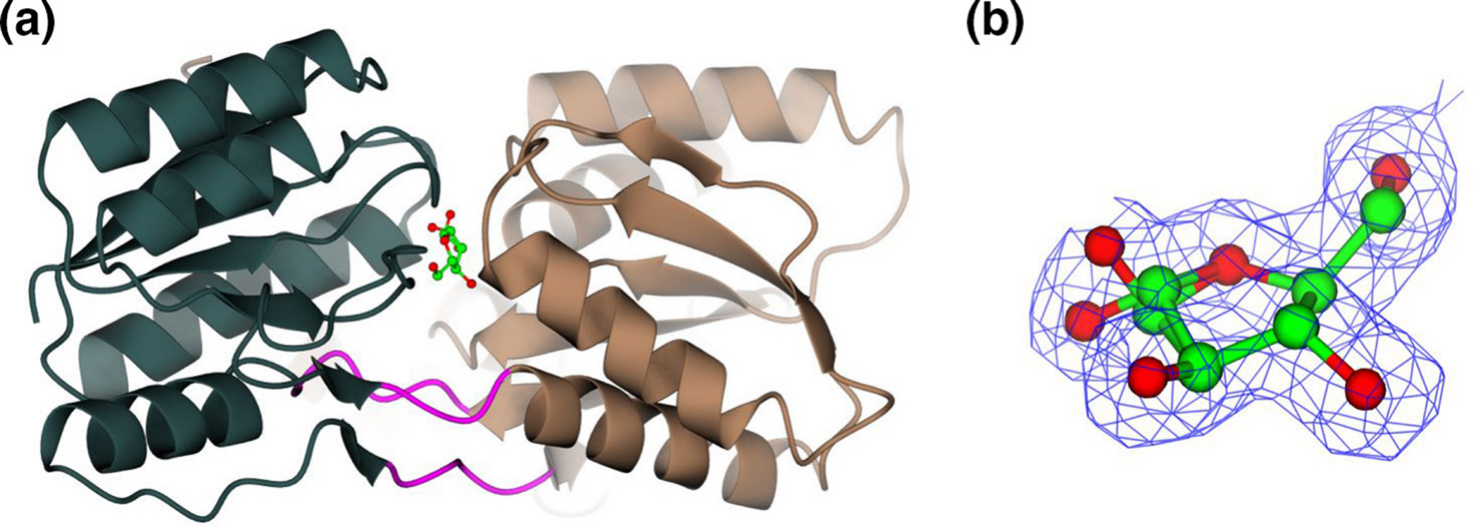
GafA_Sw_ is capable of binding both anomers of l-arabinofuranose. (**a**) GafA_Sw_ bound to l-arabinofuranose with domain I in slate grey, domain II in dark brown and the hinge region in magenta. (**b**) The combined ligands of α-l-arabinofuranose and β-l-arabinofuranose. The blue chickenwire represents the experimentally derived composite electron density map (2Fo-Fc) of the ligand contoured to 1.0 RMSD.

To explore further the molecular basis of l-arabinose selectivity in GafA_Sw_, we determined its 3D structure using X-ray crystallography. Purified GafA_Sw_ was co-crystallized with l-arabinose and the structure of the ‘closed’ ligand bound form was resolved to 1.7 Å (Fig. 4a, Table 1). Two monomers with a root-mean-square deviation (RMSD) of 0.29 Å were observed in the asymmetric unit. The structure has been deposited in the PDB as 5OCP. Like other Cluster B SBPs, the protein contains two domains, each made up of a β-sheet comprising six strands surrounded by four or six α-helices, which are held together with a typical hinge region [6, 45, 46]. The most similar proteins identified at the structural level using the DALI server were the d-galactofuranose bound GafA_Ec_ and the l-arabinofuranose and d-galactofuranose bound forms of GafA_Ms_ (Fig. S4), which using the secondary-structure matching (SSM) algorithm [37] on CCP4mg [47] displayed calculated RMSDs of 1.01, 1.08 and 1.11 Å respectively.

Upon completing the refinement of the protein structure, we identified a significant residual feature in the electron density maps in an enclosed pocket sandwiched between the two protein domains. All possible forms of l-arabinose that were present in the crystallization mix were tested but the density present in the binding site only matches to the l-Ara*f* form (Fig. 4b). Further refinement revealed the presence of both the alpha- and beta-anomers of the sugar in approximately 50 % occupancy. The accommodation of alpha- and beta-anomers of sugars is a recurring feature in SBPs first described for l-arabinopyranose binding to arabinose binding protein of *E. coli* [48] and more recently observed for d-galactose binding to GafA_Ec_ [11].

### Extensive ligand binding site similarities in GafA proteins

**Fig. 5.**
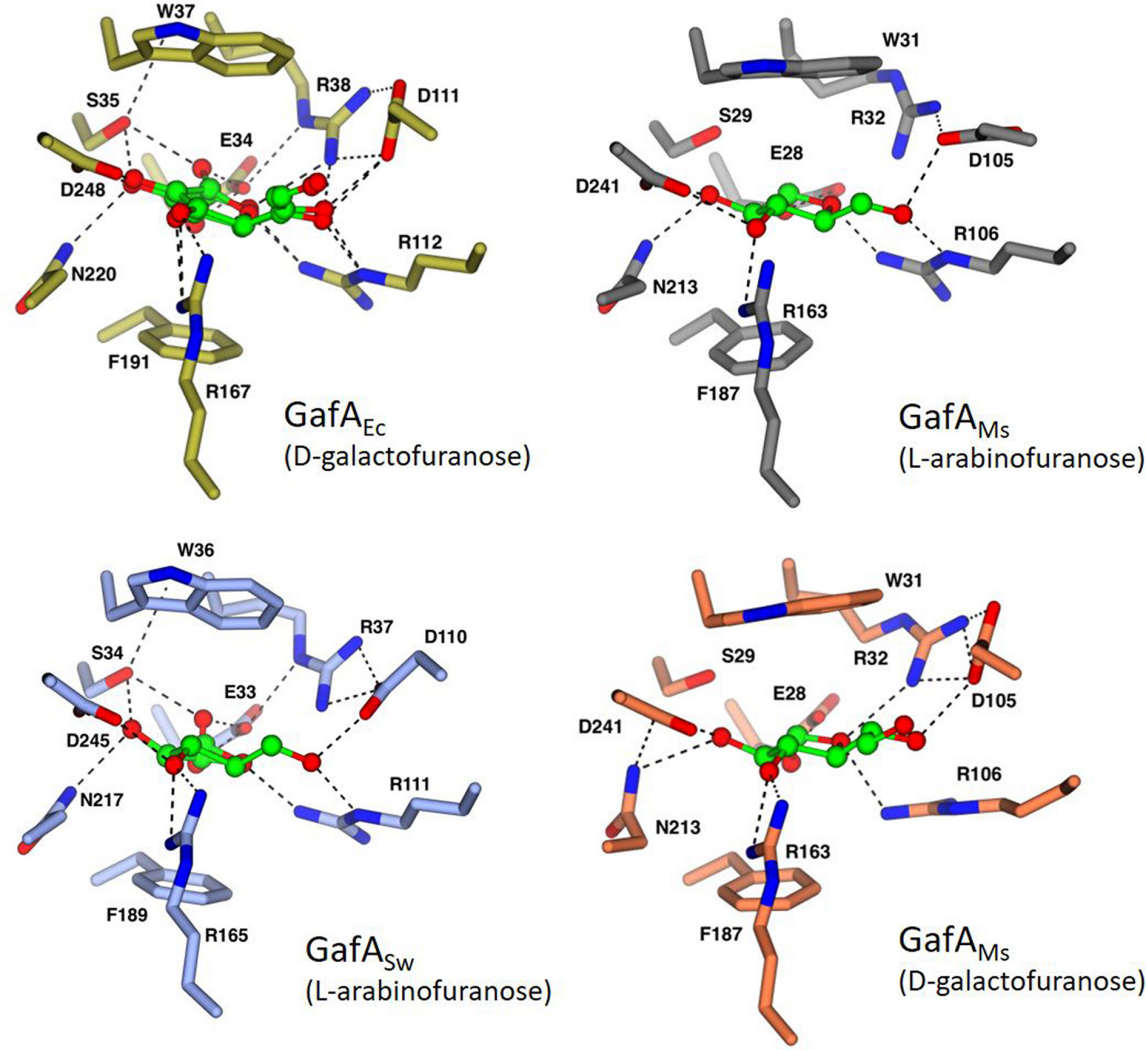
Binding site residues are identical in GafA_Ec_ (PDB: 2VK2, gold), GafA_Sw_ (PDB: 5OCP, light blue) and GafA_Ms_. Some amino acids adopt different positions in the presence of different ligands, as seen with GafA_Ms_ bound to either l-arabinofuranose (PDB: 6HBM, grey) or d-galactofuranose (PDB: 6HBD, orange).

With the structures of GafA_Ec_ and GafA_Ms_ having high levels of overall similarity to GafA_Sw_, we examined the ligand binding sites in more detail to identify commonalities and differences (Figs 5 and S6). There is remarkable conservation of all the amino acids in the binding sites of all three GafA proteins. Both the bottom and the top of the sugar ring are held through two aromatic residues, a Phe on the bottom side and a Trp on the top side, a common feature seen in other monosaccharide binding proteins [6]. Notably while the residues on the top binding surface are absolutely conserved at the sequence level, they adopt subtly different positions in the binding site and in how they coordinate the sugar.

### The positioning of a conserved aspartate appears to dictate sugar selectivity in GafA proteins

**Fig. 6.**
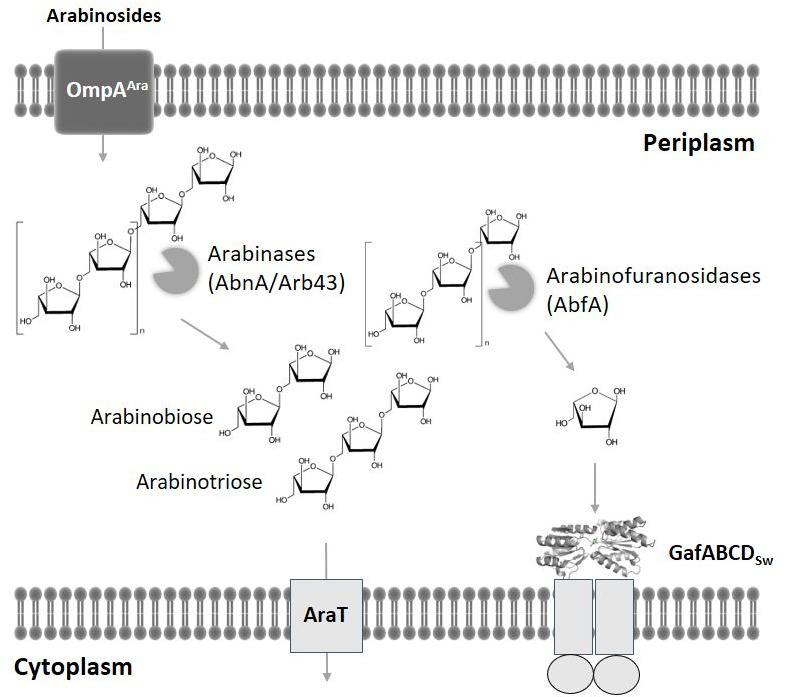
Model for arabinan utilization by *Shewanella* sp. ANA-3. The model is based on a refinement of the predictions of Rodionoc *et al*. [41] and the genes linked to each are described in the main text.

**Fig. 7.**
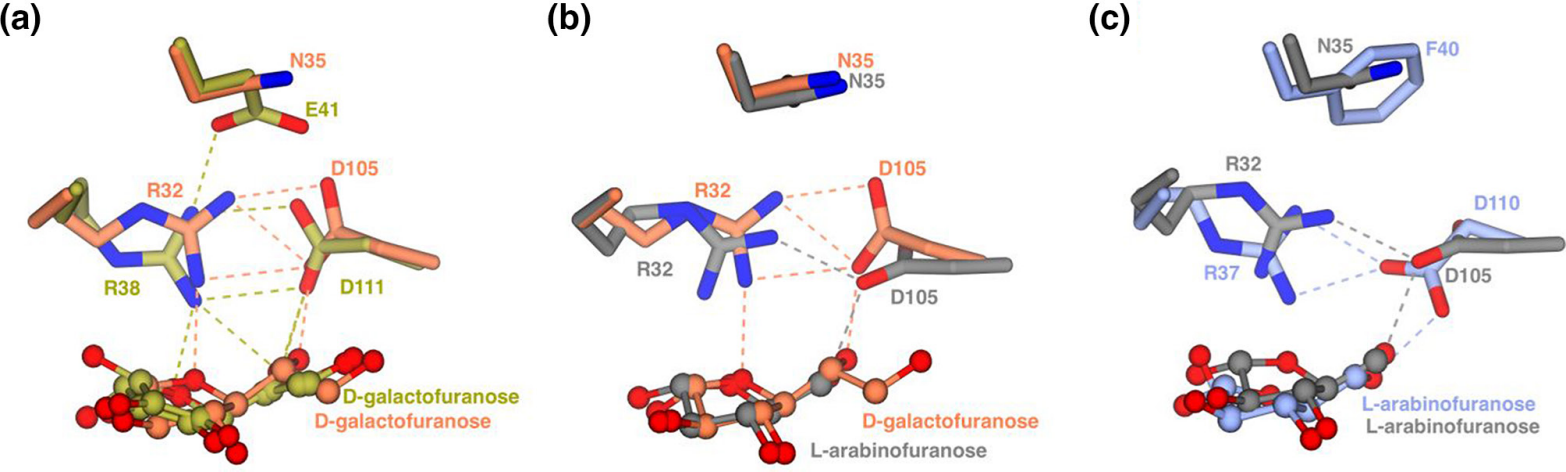
The flexibility of a ligand interacting aspartate is required for the coordination of d-galactofuranose and not l-arabinofuranose. (**a**) GafA_Ms_ (orange) and GafA_Ec_ (gold) can similarly accommodate d-galactofuranose with D105/D111 pointing away from the binding site. (**b**) GafA_Ms_ (grey: l-arabinofuranose bound, orange: d-galactofuranose bound) can also accommodate l-arabinofuranose with D105 shifting closer to the binding site. (**c**) GafA_Sw_ (light blue) can also accommodate l-arabinofuranose with D105 shifting closer to the binding site but unlike GafA_Ms_ (grey), it is unable to accommodate the larger d-galactofuranose due to the neighbouring F40.

We examined the role of the Asp residue (Asp110 in GafA_Sw_), and its equivalents in our structure and the other GafA proteins. This positioning of the residue has previously been recognized as a key determinant of sugar recognition in GafA_Ec_ as the salt bridge formed between Asp111 and Arg38 increases the size of the binding site allowing the more extended d-galactofuranose to bind over the d-galactopyranose [11]. By now having additional GafA proteins that bind furanoses of different sizes, the role of this Asp can be further assessed. It is notable that the position of this residue is the variable across the four structures shown in Fig. 6. As the GafA_Ms_ protein has been crystallized with both d-galactofuranose and l-Ara*f* it provides a missing link to help us interpret selectivity of sugar binding to the other two proteins [22]. Both the GafA_Ec_ and the GafA_Ms_ bind d-galactofuranose with the Asp in an ‘up’ position making space in the binding site to accommodate the longer sugar molecule (Fig. 7a). By comparing the two structures of GafA_Ms_ with either sugar, the coordination of the shorter l-Ara*f* requires the equivalent Asp105 to adopt a different rotamer to that seen with d-galactofuranose to preserve its charge-dipole H-bond to the sugar (Fig. 7b). This is then consistent with the position of the equivalent Asp in GafA_Sw_ (Asp110) as this also adopts a similar ‘down’ position to coordinate the shorter l-Ara*f* (Fig. 7c). While GafA_Ec_ and GafA_Ms_ can bind both sugars, GafA_Sw_ can only recognize l-Ara*f* (Fig. 3), which is possibly caused by the bulky Phe40 variant seen in GafA_Sw_ that would prevent the Asp110 from adopting multiple conformations through steric hindrance (Fig. S5). Hence, very subtle changes in the binding site are seen, which are driven by changes in the secondary shell of amino acids surrounding the binding site.

## Discussion

Bacteria have evolved a prodigious ability to scavenge nutrients from their environment, with high-affinity transporters enabling growth in environments with low (micromolar or below) concentrations of nutrients. Bacteria living in complex environments such as soil or seawater have evolved large repertoires of binding-protein-dependent transporters of the ABC, TTT and TRAP families [42, 49–51]. Predicting the specificity of binding-protein-dependent transporters is easier than for other classical secondary and other primary transporters, due to our detailed structural knowledge of the SBP subunit, and proteins have been structurally classified into families that largely sorts them into groups that bind generally similar ligands [45, 52, 53]. However, within these larger groupings there is much diversity and some classical families such as TRAP, which are commonly assumed to only bind dicarboxylates, do in fact bind a diverse range of organic acids including sugar acids, amino acids and sulphonates [44, 49, 54]. The Cluster B family of SBPs generally recognize sugars of different types and many monosaccharide-specific examples are known [45, 52]. Beyond predicting the general class of ligand, more specific predictions become more difficult [6, 53]. The discovery of GafA_Ec_ was a surprise as the protein was clearly in the monosaccharide cluster B class of SBPs and appears to be co-regulated with the known d-galactose transporter. However, while this system, the Mgl system, transports d-galactopyranose it then transpired that the bacterium makes at the same time a second ABC transporter to capture the d-galactofuranose [11]. In this study the structure of GafA_Sw_ revealed how a GafA protein, while still recognizing a furanose form of a sugar, is selective for l-Ara*f* over d-galactofuranose. At the same time the binding site residues are identical in all the GafA proteins and very highly conserved in all the other monosaccharide SBPs. Hence, this study confirms the ease of a general prediction of ligand specificity as a sugar, but reinforces that extremely subtle changes in the binding site architecture dictate the sugar selectivity. The experimentally measured *K*_D_ of 5.8±0.51 µM now needs to be corrected for the 12.5 % of substrate present in the sample of l-arabinose, giving an effective *K*_D_ of ~0.73 µM, which now being sub-micromolar is consistent with the affinity of other monosaccharide SBPs for their native ligands.

The GafA_Sm_ protein, which exhibited very similar overall binding characteristics to GafA_Ec_, is similarly not encoded with other linked catabolic genes, although its expression is known to be induced by l-arabinose and d-fucose [42]. Our study corroborated the above results, as both of these sugars increased the thermal stability of GafA_Sm_ but we also demonstrated d-galactose binding. Interestingy, the GafA_Sm_ orthologue from *Rhizobium leguminosarum* bv. *viciae* 3841, namely RL2376, was found to bind d-galactose and its use as a biosensor was proposed [55], although it is highly likely to also bind l-arabinose and might not be particularly selective. Another study found that the GafA_Sm_-encoding operon was expresed in response to desiccation induced by high NaCl in *Sinorhizobium meliloti* 1021 [56], and a known response to this is the production of galactose-containing oligosaccharides [57], consistent with a function of scavenging d-galactose to provide precursors for these glycans.

The use of the powerful bioinformatics tools MicrobesOnline and RegPrecise [24, 40], combined with the experimental fitness data included in MicrobesOnline [25], led us to a strong hypothesis that the GafA_Sw_ protein was probably functioning in the context of l-Ara*f* uptake, which was borne out in our data and even more so in the unique specificity of this GafA protein for l-arabinose over d-galactose. Given this finding, a closer examination of the other genes in this extensive gene cluster enables a reconstruction of the possible function of the genes contained within it in the use of arabinan as a carbon source in the soil environment (Fig. 6). This includes a possible outer membrane porin for arabinosides (Shewana3_2085), a series of secreted and periplasmic arabinases and periplasmic and cytoplasmically located arabinofuranosidases (Shewana3_2067, 2069, 2077, 2078, 2082, 2086), the Gaf transporter (Shewana3_2077–76) along with other putative secondary carriers (Shewana3_2081 and Shewana3_2084), followed by the intracellular catabolic pathways shown in Fig. 1(b). In fact, 13 genes in this cluster are essential for growth on l-arabinose [25].

It is of interest that unlike *Sinorhizobium meliloti* and *E. coli *, an l-arabinopyranose (AraFGH) transporter is not present in the cluster or in the entire genome of *Shewanella *, suggesting that *gafABCD* encodes the sole l-arabinose transporter in this organism, which would be consistent with the strong growth phenotype. As far as we are aware, this would be unique in any bacterium and probably suggests that selective advantage of the immediate uptake of periplasmically liberated l-Ara*f* in the oligosaccharides that are being consumed by the bacterium. While the rate of spontaneous conversion of the released l-Ara*f* to l-arabinopyranose is likely to be in the range of minutes, data on this in physiological conditions are not available to our knowledge. From our previous work on GafA_Ec_ when we released the d-galactofuranose bound to the protein in the presence of DMSO, which slows the rate of interconvertion, we were able to show significant furanose form in solution by NMR immediately after release, but a few hours later this was at equilibrium levels [11]. In summary, using a combination of published phenotypes, bioinformatics, biochemistry and structural biology we have expanded our knowledge of bacterial Gaf-type ABC transporters and discovered a system that has evolved selectivity for l-Ara*f* over d-galactofuranose. For the design and engineering of bacteria with enhanced plant biomass-degrading capabilities, this gene cluster, with its unique Gaf system, could be considered as useful targets for improving the capabilities of chassis strains for use in industrial biotechnology.

## Data Summary

The data that support the findings of this study are publicly available in the RCSB PDB database under the PDB identifier = 5OCP (i.e. crystal structure of Shewana3_2073). Raw data that support the findings of this study are available from the corresponding author, upon request.

## Supplementary Data

Supplementary material 1
