# Supplementary material for: Characterization of the l-arabinofuranose-specific GafABCD ABC transporter essential for l-arabinose-dependent growth of the lignocellulose-degrading bacterium Shewanella sp. ANA-3
Source: Microbiology (Reading). 2023 Mar 15;169(3):001308. doi: 10.1099/mic.0.001308 (PMC10191376; doi:10.1099/mic.0.001308)
Supplement: Supplementary material 1 [file mic-169-1308-s001.pdf]

Supplementary Figures for Droutis *et al*., (2023) Characterisation of the L-arabinofuranose-specific GafABCD ABC transporter essential for L-arabinose dependent growth of the lignocellulose degrading bacterium *Shewanella* sp. ANA3

A)

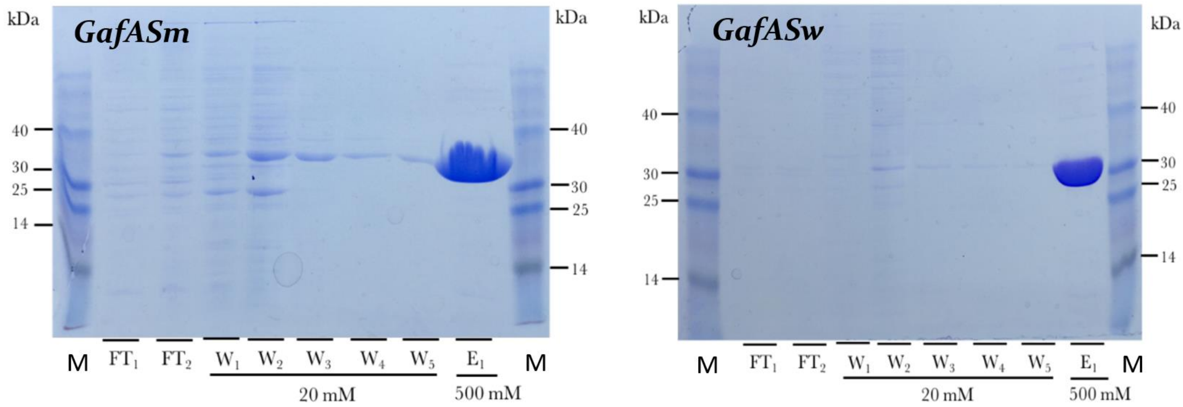

B)

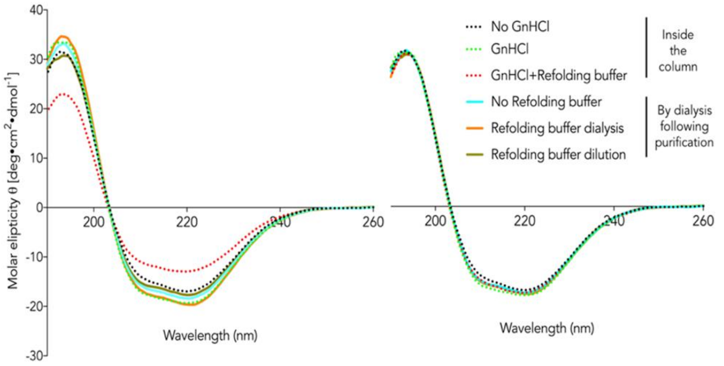

Fig. S1. A) Purification of the two GafA proteins. SDS-PAGE analysis of TSE-extracted GafA proteins (GafASm left panel, GafSw right panel) following Ni^{2+}-affinity purification. FT = flow through, W = wash, E = elution (at indicated imidazole concentrations). M = markers (indicated masses of selected bands are shown). **B)** ***Far UV circular dichroism spectra of GnHCl pretreated GafASm.*** CD analysis was performed on GafASm following treatment with GnHCl denaturant for the release of any prebound galactose. The GnHCl treatment happened either during the $Ni2^{+}$-based purification run, with or without the presence of refolding buffer (dotted line); or by overnight dialysis with 2M GnHCl (continuous line), followed by dialysis (orange) or dilution (olive green) in refolding buffer. The blank-corrected data are plotted in the spectra shown on the **left**, whereas the normalized data correcting for protein concentration are shown on the **right**. The analysis shows that all fractions retain their secondary structure elements intact, indicating that the full fraction of GafASm refolds back to its mature conformation following treatment by GnHCl. The obtained spectra are similar to CD plots of proteins rich in α-helices *ie*. a common feature of SBPs. The run was performed on J-810 spectropolarimeter with 6 μM of GafASm in 50 mM NaF and 20 mM Tris-HCl (pH=7.5). Graph was plotted in GraphPad Prism 7.0.

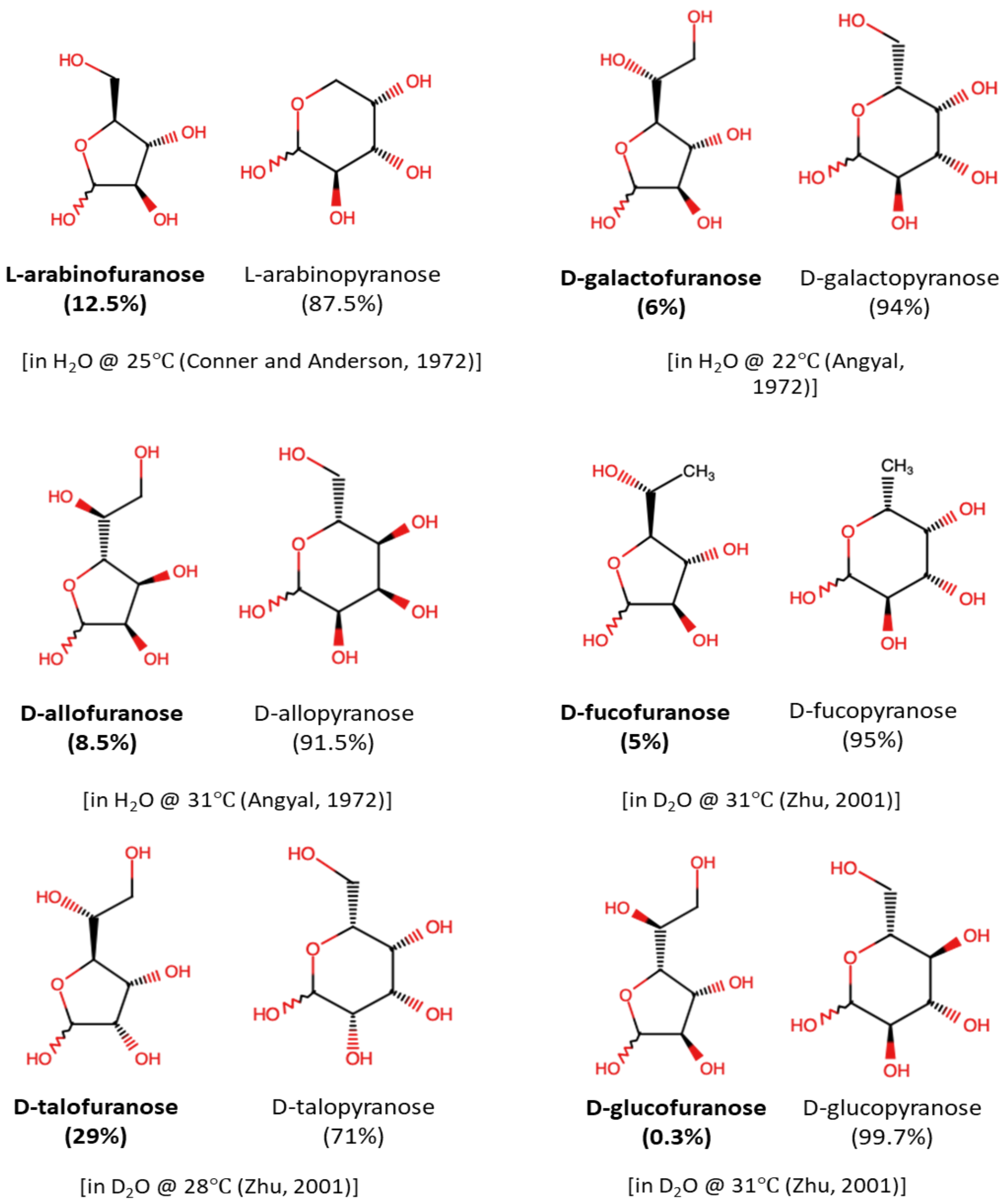

Fig. S2. The resting equilibrium ratios of common monosaccharides. The sources of these are Conner & Anderson (1972) *Carbohydrate Research* 25:107-116 DOI:10.1016/S0008-6215(00)82751-2, Angyal *et al*., (1972) *Australian Journal of Chemistry* 25:1695-1710 DOI:10.1071/CH9721695 and Zhu, Zajicek & Serianni (2001) J Org Chem 66(19):6244-51. DOI: 10.1021/jo010541m.

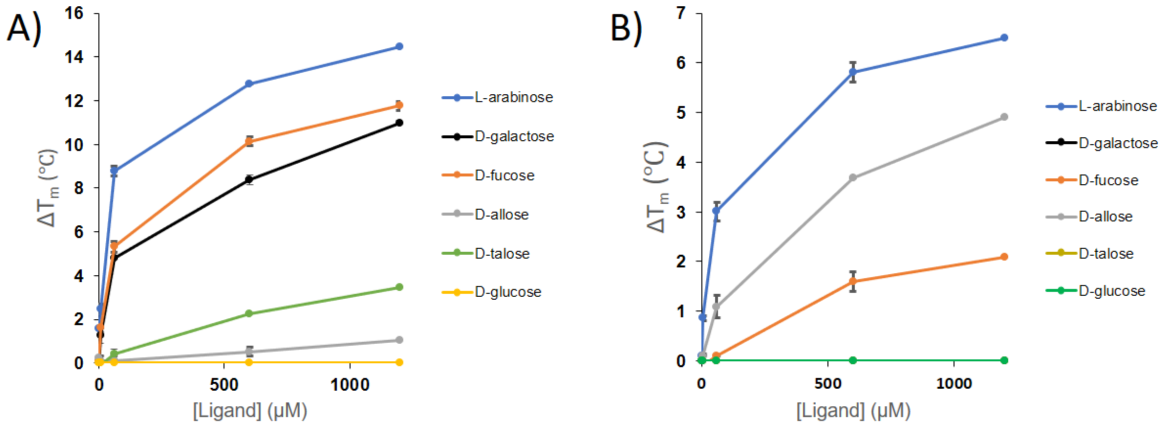

Fig. S3. Analysis of ligand binding using DSF for A) $GafA_{Sm}$ and B) $GafA_{Sw}$ up to 1.2 mM ligand.

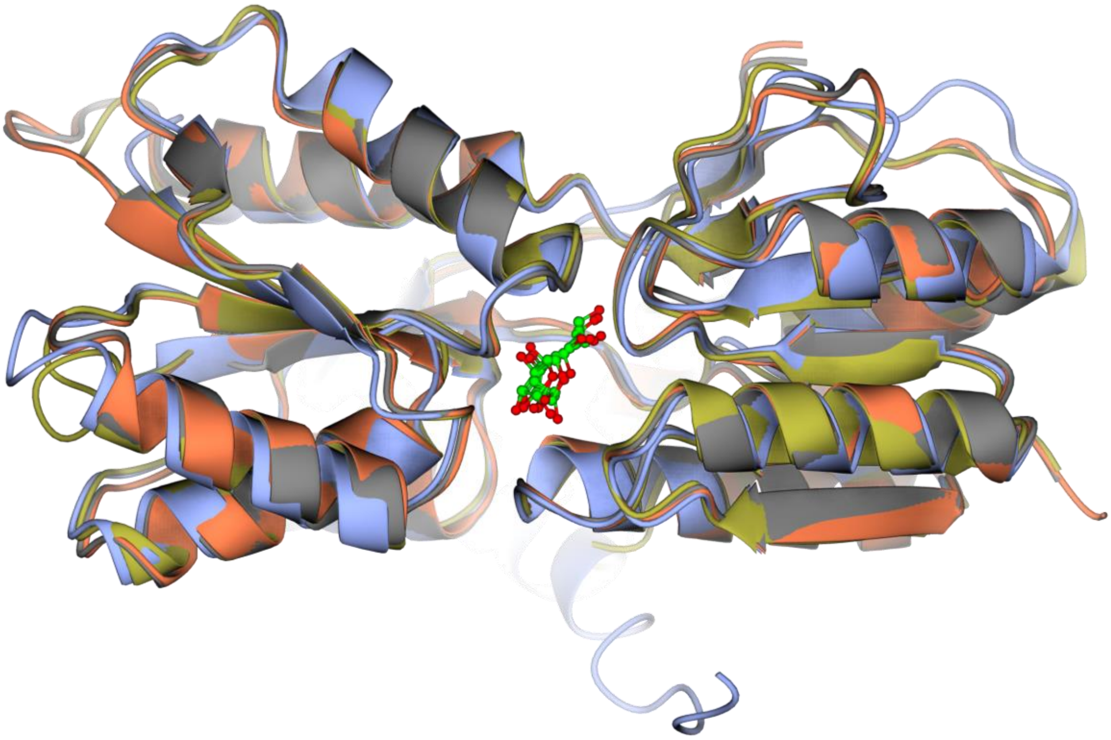

Protein Name	PDB ID	Ligand Bound	RMSD (with $GafA_{Sw}$)
$GafA_{Sw}$	5OCP (this study)	L-arabinofuranose	-
$GafA_{Ms}$	6HBM (Li *et al.,* 2019)	L-arabinofuranose	1.08 Å
$GafA_{Ec}$	2VK2 (Horler *et al.,* 2009)	D-galactofuranose	1.01 Å
$GafA_{Ms}$	6HBD (Li *et al.,* 2019)	D-galactofuranose	1.14 Å

Fig. S4. The overall structure alignments of GafAs using $GafA_{Sw}$ as the fixed model. Inset Table show the RMSD (root mean square distance) calculated corresponds to the alignment of the respective structures with that of $GafA_{Sw}$.

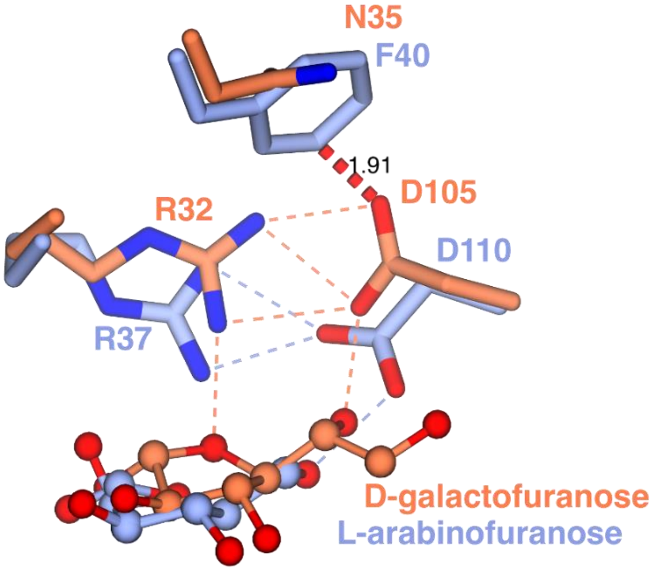

Fig. S5. Comparison of the $GafA_{Sw}$ protein (light blue) binding L-arabinofuranose and the $GafA_{Ms}$ protein (orange) binding D-galactofuranose, highlighting a potential steric clash that would likely occur in the $GafA_{Sw}$ protein if D-galactofuranose was positioned in the binding site in the same position as seen in $GafA_{Ms}$, highlighting the possible role F40 in constraining the ligand binding specificity.

	30	-	33	34	35	36	37	-	40	-	62	-	110	111	-	129	-	132	-	156	-	159	160	161	-	165	-	189	-	216	217	218	219	-	244	245	-	247	-	265
$GafA_{Sw}$	V		E	S	G	W	R		F		Q		D	R		F		E		T		A	T	A		R		F		H	N	D	E		V	D		V		L
$GafAE_{c}$	V		E	S	G	W	R		E		Q		D	R		N		E		T		A	S	V		R		F		H	N	D	D		I	D		V		L
$GafA_{Ms}$	V		E	S	G	W	R		N		G		D	R		F		E		T		A	D	P		R		F		Q	N	D	D		V	D		T		C

Fig. S6. Sequence alignment of $GafA_{Sw}$, $GafA_{Ec}$ and $GafA_{Ms}$. Residues within 7Å of the L-arabinofuranose binding site in $GafA_{Sw}$ were aligned with that of $GafA_{Ec}$ and $GafA_{Ms}$. Highlighted in green are residues that are thought to interact with L-arabinofuranose in $GafA_{Sw}$ and the corresponding residues in the aligned sequences. The secondary shell residue Phe40 which is thought to modulate ligand binding in $GafA_{Sw}$ and the corresponding aligned residues are highlighted in blue.
